# Technical Aspects: Coronary Artery Bypass Grafting in a Case of Dextrocardia With Situs Inversus

**DOI:** 10.7759/cureus.14932

**Published:** 2021-05-10

**Authors:** Mithun Sundararaaja Ravikumar, Vijayanand Palanisamy, Mugundhan Tamilselvan, Sureshkumar Sangili, Rajan Sethuratnam

**Affiliations:** 1 Department of Cardiothoracic Surgery, Madras Medical Mission Hospital, Chennai, IND

**Keywords:** dextrocardia, situs inversus, bypass grafting, internal mammary artery, saphenous vein

## Abstract

Dextrocardia with situs inversus is a rare congenital anomaly in which the heart and the abdominal organs orient themselves in a mirror-image reversal of the normal anatomy. Coronary artery disease incidence is similar to that of the normal population. Performing coronary artery bypass grafting in this subset of the population poses few difficulties. These limitations can be overcome by few technical adjustments by the surgeon and the team which will be discussed in our article.

## Introduction

Dextrocardia with situs inversus is an infrequent clinical condition having an average incidence of 1:100,000 [1]. The incidence of coronary artery disease (CAD) does not differ from that of the normal population [2]. The anatomy of this condition requires few adjustments during the conduct of surgery. In dextrocardia, the surgeon will operate from the left side of the patient in most centers. However, surgery can also be performed by the surgeon in the conventional position. In this article, we will discuss the technical aspects of how we successfully performed coronary artery bypass grafting (CABG) conventionally.

## Case presentation

**Figure 1:**
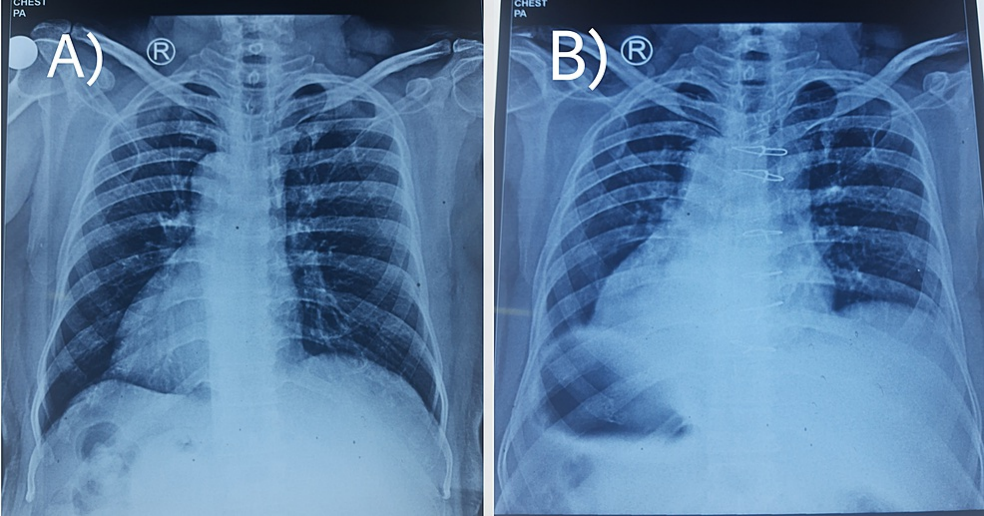
(A) Chest roentgenogram showing dextrocardia. (B) Chest roentgenogram showing situs inversus.

A 59-year-old gentleman came to our institution with a complaint of chest pain for a week. On evaluation, his chest roentgenograms revealed that the apex of the heart was on the right side of the chest with a gastric fundus shadow on the right side (Figures 1A, 1B).

His echocardiogram confirmed dextrocardia with normal biventricular function. Coronary angiogram revealed CAD with lesions in the left anterior descending (LAD), diagonal, ramus intermedius, and posterior descending arteries (PDA). Elective CABG was planned for the patient.

**Figure 2:**
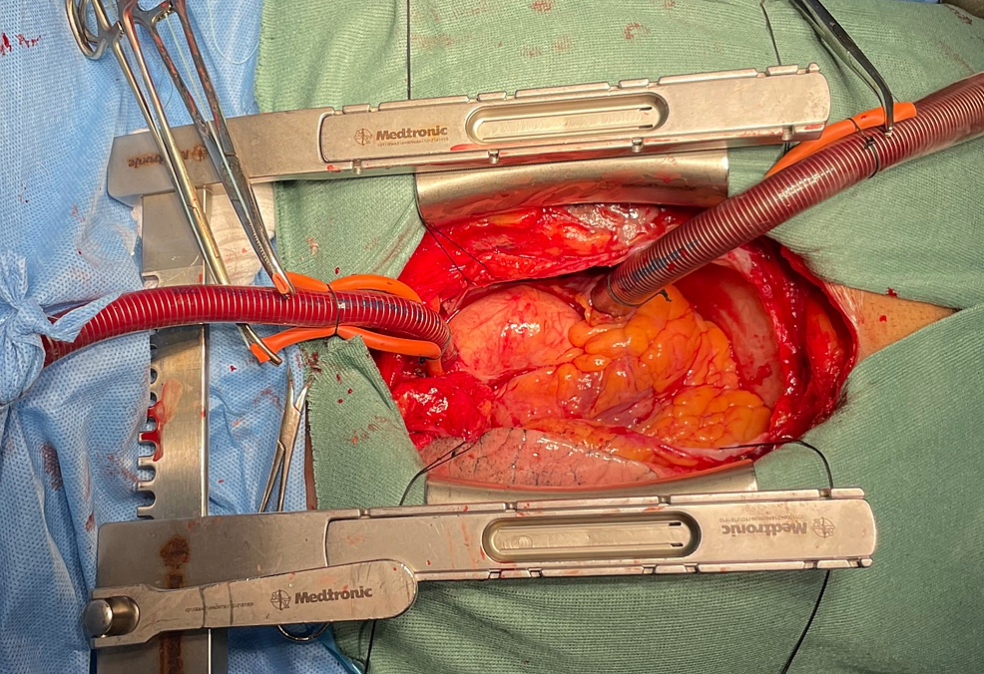
Cardio-pulmonary bypass instituted by aortic and right atrial cannulation.

A median sternotomy was performed. The right internal mammary artery (RIMA) and great saphenous vein were harvested. After systemic heparinisation, the cardio-pulmonary bypass (CPB) was instituted by aortic and right atrial cannulation. Moderate hypothermia of around 32 degrees Celsius was maintained. Aorta was cross-clamped. The heart was arrested using a blood cardioplegic solution and repeated every 20 minutes (Figure 2).

**Figure 3:**
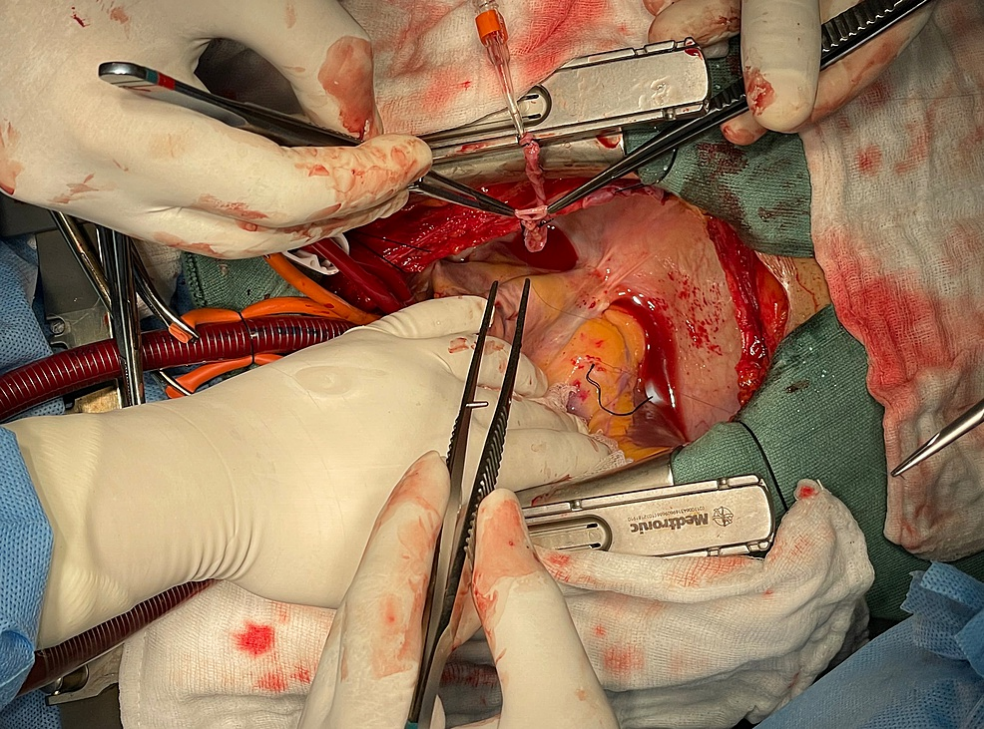
Exposure of the posterior descending artery.

The surgeon was operating from the conventional right side of the patient. The first conduit to be anastomosed distally was the saphenous vein to the PDA. The assistant who was on the left side of the surgeon (third assistant) retracted the acute margin of the heart (located on the left side) using the left hand with gauze for exposure of the PDA (Figure 3).

**Figure 4:**
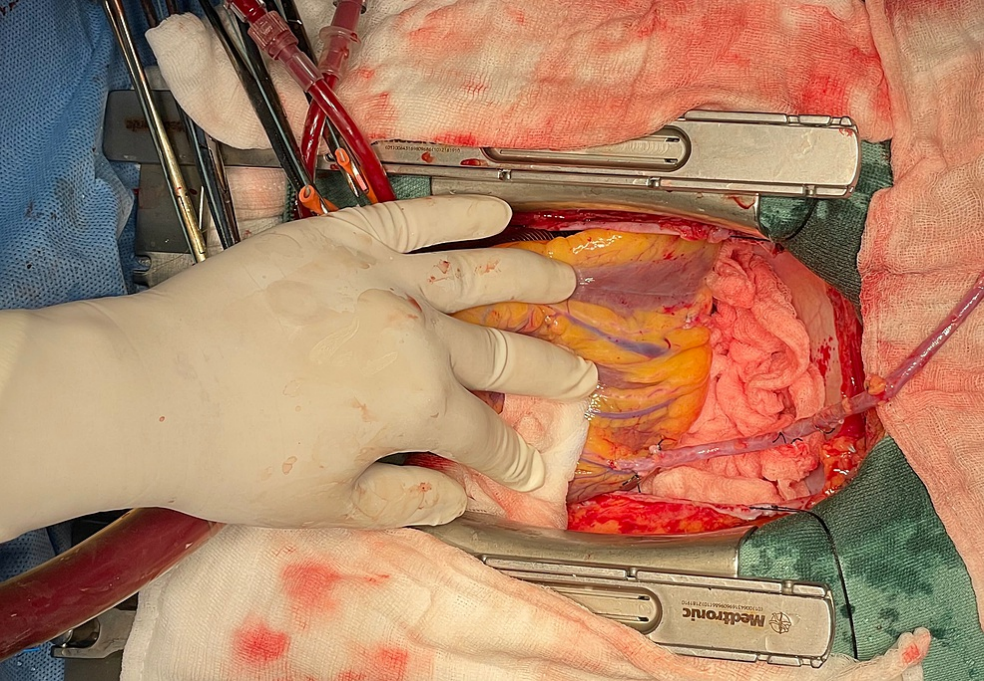
Exposure of the ramus intermedius artery.

The next vessels which were anastomosed with the saphenous vein grafts were diagonal and ramus intermedius arteries. The surgeon placed a sponge gauze under the base of the heart. The third assistant retracted the obtuse margin of the heart for exposure of the diseased ramus intermedius artery (Figure 4).

**Figure 5:**
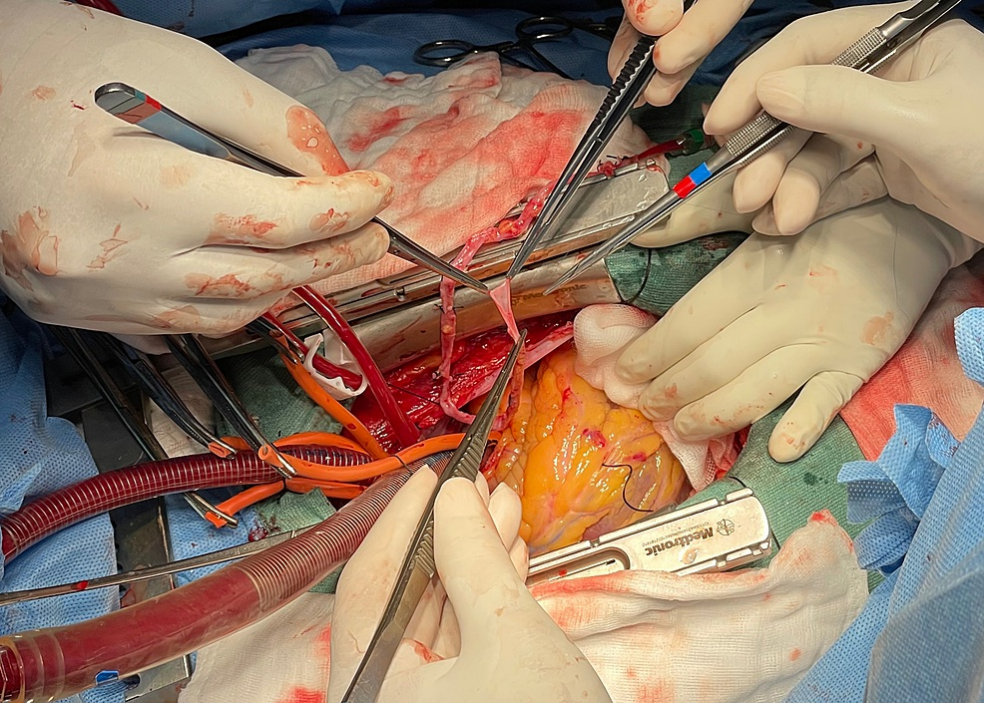
Exposure of the diagonal artery.

The second assistant will position the heart using his left hand for the exposure of the diagonal artery (Figure 5).

**Figure 6:**
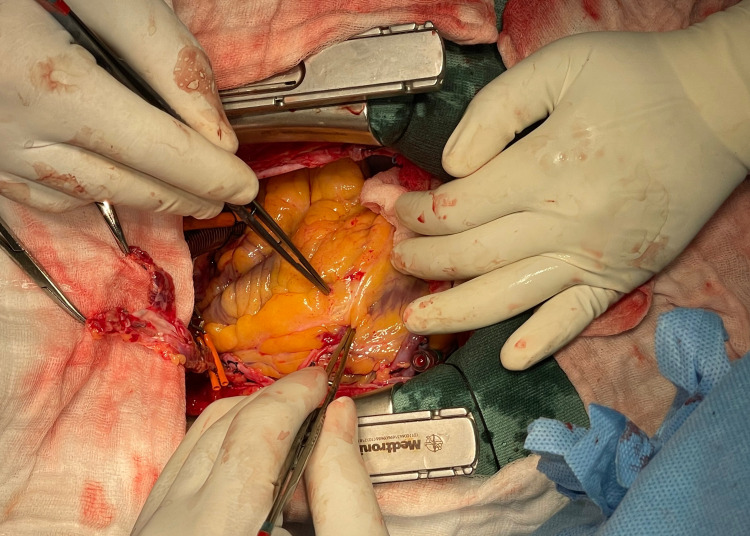
Exposure of the left anterior descending artery. The harvested right internal mammary artery was positioned towards the head end of the patient for anastomosis.

Finally, the harvested RIMA was then prepared for distal anastomosis to the LAD. We created a pericardial window on the right side and tunneled the RIMA through the window. The first assistant positioned the RIMA towards the head end for easier anastomosis and parachuting off the conduit to the LAD (Figure 6).

**Figure 7:**
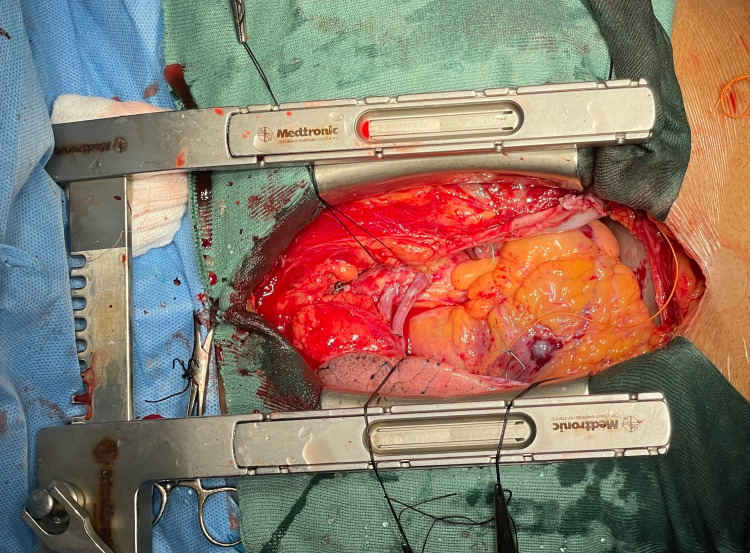
Completion of the proximal anastomosis of vein grafts onto the ascending aorta.

Once the distal anastomosis was completed, rewarming was initiated. The aortic cross-clamp was removed. A partial clamp was applied to the ascending aorta and the proximal anastomosis of the vein conduits was performed onto the aorta (Figure 7).

Heart regained its sinus rhythm gradually. On full rewarming, we came off CPB gradually. Routine chest closure was performed.

## Discussion

Dextrocardia is an uncommon cardiac anomaly that was first recounted by Hieronymus Fabricius (1606) in which the heart is positioned in the right hemithorax with its base to apex axis directed towards the right side. CAD occurs in the same proportion as that of the normal population. The first CABG in dextrocardia was performed in 1980 [3].

While performing surgery, many surgeons prefer standing to the left side of the patient for convenience during anastomosis. In this article, we have discussed that it is possible to perform in the conventional manner where the surgeon stands right to the patient [4].

RIMA is usually harvested instead of the left internal mammary artery (LIMA) in these cases. In our case, the only time the surgeon positioned himself to the left of the patient was while harvesting RIMA.

LIMA is usually avoided since the length of the graft will not be adequate to anastomose to the LAD resulting in the stretch of the graft and inadequate flow. The LIMA will have to cross the midline exposing it to high chances of injury during the re-do surgery. The LIMA flow may get compromised due to marked angulation at its origin leading to a dismal result of surgery. These limitations are avoided by using RIMA.

Other considerations before incision include placement of electrocardiogram electrodes over the right hemithorax and placement of a central venous catheter in the left-sided internal jugular vein which will be monitored by the cardiac anesthetist.

## Conclusions

Dextrocardia with situs inversus is a rare clinical condition presenting itself as a mirror image of the normal anatomic orientation. CAD in this subset of patients is similar to that of the normal population. Performing CABG in a dextrocardia with situs inversus appears to be challenging but few minor adaptations by the surgeon and the team will make the procedure easier to perform.

## References

[1] Rosenberg HN, Rosenberg IN (1949). Simultaneous association of situs inversus, coronary heart disease and hiatus hernia; report of a case and review of literature. Ann Intern Med.

[2] Subash S, Simha PP, Manjunatha N (2017). Off-pump coronary artery bypass surgery in a patient with dextrocardia and situs inversus: anesthetic, surgical consideration and role of transesophageal echocardiography. Heart Views.

[3] Irvin RG, Ballenger JF (1982). Coronary artery bypass surgery in a patient with situs inversus. Chest.

[4] Saad RA, Badr A, Goodwin AT, Dunning J (2009). Should you stand on the left or the right of a patient with dextrocardia who needs coronary surgery?. Interact Cardiovasc Thorac Surg.

